# High- and ultrahigh-spatial-resolution geochronology

**DOI:** 10.1093/nsr/nwaf200

**Published:** 2025-05-20

**Authors:** Qiu-Li Li, Hao Wang

**Affiliations:** State Key Laboratory of Lithospheric Evolution, Institute of Geology and Geophysics, Chinese Academy of Sciences, China; College of Earth and Planetary Sciences, University of Chinese Academy of Sciences, China; State Key Laboratory of Lithospheric Evolution, Institute of Geology and Geophysics, Chinese Academy of Sciences, China; College of Earth and Planetary Sciences, University of Chinese Academy of Sciences, China

Over 100 years ago, the law of radioactive isotope decay established the foundation for isotope geochronology, enabling the construction of a timeline for geological evolution. The complexity of geological evolution means that the record of a specific geological event may be confined to a particular mineral grain, or even a small portion of it. Modern geochronology has progressed through a gradual reduction in sample size, transitioning from the analysis of whole rocks and multigrain fractions to single mineral grains and grain fragments. Benefitting from the technological breakthroughs in secondary ion mass spectrometry (SIMS), laser ablation multi-collector inductively coupled plasma mass spectrometry (LA–(MC)–ICPMS) and atom probe tomography (APT), the past five decades have witnessed a rapid development from solution-based bulk analysis to solid-based microanalysis. These advances have culminated in high-spatial-resolution methods capable of determining isotope ratios from tiny minerals or within individual zones of crystals. We focus on the fundamental capabilities that enable high-spatial-resolution methods in geochronology, which includes U–Pb and β-decay isotopic dating.

**High-spatial-resolution U–Pb geochronology.** Rock-forming and accessory minerals commonly exhibit zoning in their elemental and isotopic compositions, including variations in age within geochronometric phases. These zonation patterns result from three principal mechanisms: (i) the incorporation of preexisting material (‘inherited’ components), (ii) the dynamic chemical evolution of parent magmas or fluids during mineral growth and (iii) the post-crystallization processes such as diffusion, metasomatism or radiation damage that can partially reset isotopic systems. Such zoning can be resolved and spatially characterized by using optical microscopy combined with high-resolution compositional mapping techniques, such as cathodoluminescence or backscattered electron imaging. These methods facilitate targeted microanalysis by identifying and avoiding secondary features (e.g. fractures, inclusions or lattice defects) that may compromise age accuracy. By integrating real-time or pre-analysis imaging with microbeam sampling, researchers can selectively interrogate domains with the wealth of textural information available—essential for interpreting chronological results in samples that represent a complex mixture of materials from various sources [1].

**Figure 1.**
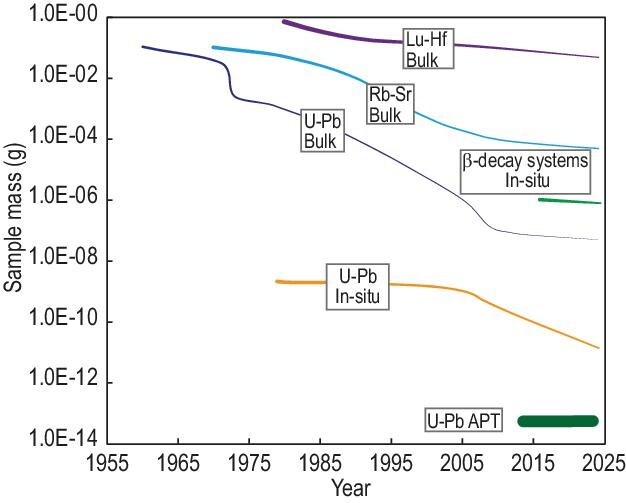
Reduction in sample consumption brought about by technological progress. The thickness of the lines represents the relative magnitude of the corresponding age uncertainties that the technique can achieve.

The U–Pb isotopic dating system has emerged as one of the most pivotal geochronological tools due to its unique advantages, including the suitable half-lives of uranium isotopes (²³⁸U and ²³⁵U), the abundance of datable minerals (e.g. zircon, monazite and baddeleyite) and high closure temperatures that preserve isotopic integrity during thermal events. Additionally, the high ionization efficiency of Pb and the significant mass difference between parent (U) and daughter (Pb) isotopes facilitate high-precision mass spectrometric analyses, particularly for microscale *in situ* applications. Since the 1970s, U–Pb techniques have undergone transformative technological advancements to achieve high-precision age measurements with minimal sample consumption [2]. Recent innovations in micro-sampling methods, such as laser ablation and focused ion beam milling [3], have spatially resolved *in situ* analyses while minimizing sample destruction. Since the 1980s, the development of SIMS techniques has marked a significant milestone by enabling age determinations at spatial resolutions of ∼10 μm. Further refinements in ion-source design have enhanced spatial resolution to <3 μm—a critical capability for analysing precious extraterrestrial samples, such as lunar materials, for which sample volumes are limited [4]. Breakthroughs in depth-profiling techniques by using SIMS and LA–(MC)–ICPMS have pushed spatial resolution to the sub-micron scale (∼100 nm), enabling the coupled age-composition analysis of mineral domains affected by complex thermal histories [5]. After 2014, the APT technique emerged as a potential tool for nanoscale geochronology by enabling direct imaging of radiogenic Pb clusters within minerals (Fig. 1) [6]. Through quantification of Pb isotope ratios in nanoscale domains (2–50 nm), this high-spatial-resolution approach may provide insights into primary crystallization ages and possible Pb loss or redistribution events, albeit with limited chronological precision.

However, the pursuit of higher spatial resolution inevitably trades off against analytical precision. While bulk analyses routinely achieve uncertainties of ∼0.1%, micron-scale SIMS and laser ablation inductively coupled plasma tandem mass spectrometry (LA–ICP–MS) analyses typically yield 1%–2% precision. At the nanoscale, APT-based U–Pb dating currently faces precision limitations of >10%, reflecting challenges in counting statistics and Pb background correction. In summary, U–Pb geochronology has evolved from bulk mineral analyses into a multidimensional tool capable of resolving geological processes across various scales—from planetary formation recorded in extraterrestrial samples to nanoscale metamorphic overprints. Future advancements in instrumentation and data-reduction algorithms will bridge the remaining gap between spatial resolution and analytical precision, approaching the ultimate physical limit set by the number of radiogenic atoms in the sample. This progress will further establish U–Pb dating as an indispensable chronometer in Earth and planetary sciences.

***In situ* dating achievements for β-decay isotopic systems.** Due to the limitations of commonly used SIMS and LA–ICP–MS in resolving isobaric interferences in β-decay isotopic systems, in which the parent and daughter isotopes possess the same number of nucleons, there has been limited advancement in analytical techniques for commonly used β-decay geochronology, such as Rb–Sr, Lu–Hf, Re–Os and K–Ca. Although micro-drilling techniques have been employed to sample certain minerals (e.g. mica and garnet) at millimeter to submillimeter scales for Rb–Sr or Lu–Hf dating [7], ion-exchange chemical separation is still required prior to final mass spectrometry measurements.

The advent of LA–ICP–MS/MS has provided an unprecedented method for *in situ* dating of β-decay radiogenic isotope systems (Fig. 1). This technique employs a cell sandwiched between two quadrupoles that can accommodate reactive gases (e.g. N_2_O, O_2_, H_2_, NH_3_, CH_4_ and SF_6_) to shift the ions of daughter isotopes to higher masses [8–10]. *In situ* β-decay geochronology using LA–ICPMS/MS not only significantly improves spatial resolution compared with micro-drilling sampling techniques and solution-based bulk analysis—achieving laser diameters ranging from 50 to 150 μm, depending on the daughter isotope content of the analysed materials—but also minimizes sample preparation. This efficiency allows rapid dating with a high sample throughput. The Rb–Sr system is the earliest developed and most successful *in situ* β decay geochronology method [8], capable of producing geologically meaningful results with an uncertainty of <3% for Rb-rich minerals, including mica, K-feldspar, illite and glauconite. This expands the application of Rb–Sr dating across a wide range of geological progresses, such as sedimentation, faulting, low-grade metamorphism, hydrothermal alteration and mineralization, which were previously challenging to be accurately constrained. Because Rb and K have similar geochemical behaviors and ionic radii, the minerals suitable for Rb–Sr dating also have the potential for K–Ca dating. Both the Rb–Sr and K–Ca systems in most rock-forming minerals possess low closure temperatures (<500°C), making them susceptible to disturbance from later alteration. *In situ* Rb–Sr and K–Ca dating techniques can effectively avoid altered domains or inclusions within minerals, thereby providing geologically meaningful ages. *In situ* K–Ca geochronology has to face an additionally analytical challenge, namely the significant isobaric interference of ^40^Ar^+^ produced in the ICP, which places it at a disadvantage compared with *in situ* Rb–Sr dating. This limitation restricts the application of *in situ* K–Ca geochronology [11]. *In situ* Lu–Hf geochronology is one of the rapidly evolving techniques developed over the past several years [9,10]. It offers a cost-effective method for dating Lu-rich minerals, with an analytical uncertainty of ∼3%–10%. Theoretically, the Lu–Hf system exhibits higher closure temperatures compared with the U–Pb, K–Ca and Rb–Sr systems for most minerals. On the one hand, *in situ* Lu–Hf dating has the advantage of revealing the crystallization age of the rocks that have undergone polyphase metamorphic overprints or hydrothermal alteration. On the other, combining *in situ* Lu–Hf and U–Pb dating with low-temperature fission track dating on minerals (e.g. apatite) can provide a comprehensive thermal history of their host rocks. Unlike the U–Pb, K–Ca and Rb–Sr systems, lutetium and hafnium have similar condensation temperatures, which results in significantly weaker elemental fractionation effects during LA–ICP–MS/MS and thus minimal dependence on matrix-matched reference materials. The *in situ* Lu–Hf dating technique opens up a new avenue for dating rare minerals (e.g. samarskite, euxenite and aeschynite) that are often too small to be separated for conventional solution-based bulk analysis and cannot be dated by using *in situ* U–Pb analysis due to the lack of matrix-matched reference materials. More importantly, garnet—the principal mineral for elucidating the chemical and thermal histories of metamorphism—is particularly suitable for Lu–Hf dating. The *in situ* technique can resolve the age zonation within single garnet crystals and directly relate the age to the recovered *P*–*T* conditions. This advancement is invaluable for enhancing our understanding of metamorphic processes [12]. It is important to note that garnet crystals exhibit variable Lu contents, showing a general decreasing trend: >100 ppm in pegmatite garnet, <10 ppm in garnet from felsic and metapelitic rocks, <5 ppm in garnet from mafic metamorphic rocks, and <1 ppm in garnet from peridotites. Currently, only garnet crystals with Lu contents of >5 ppm and formation ages of >1000 Ma can provide geologically meaningful *in situ* Lu–Hf ages with analytical uncertainties of <5%. The timing of sulfide ore deposits can be directly dated by using Re–Os geochronology; however, currently, only molybdenite and certain pyrite samples with high Re content can yield reliable *in situ* Re–Os ages through LA–ICP–MS/MS with an analytical uncertainty of <10% [13]. The potential decoupling of Re–Os presents an additional challenge for the *in situ* technique. Overall, LA–ICP–MS/MS not only offers significant advantages in analysing small and precious samples, as well as normal-sized samples with complex zonation, but also enables the simultaneous measurement of trace elements, providing additional geochemical information for the obtained ages. As a promising technique, further development is necessary to enhance the effectiveness of *in situ* β-decay dating. This includes, but is not limited to: (i) exploring novel reaction gases or combinations of gases for more efficient online element separation; (ii) developing new methods that are suitable for tandem multi-collector ICPMS, which demonstrates significantly higher sensitivity and stability; (iii) establishing *in situ* La–Ba dating for light-rare-earth-element-rich minerals such as monazite and bastnaesite; and (iv) developing reference materials that are homogeneous in their isotopic/elemental ratios or ages.
